# Meta-analysis of peripheral blood gene expression modules for COPD phenotypes

**DOI:** 10.1371/journal.pone.0185682

**Published:** 2017-10-09

**Authors:** Dominik Reinhold, Jarrett D. Morrow, Sean Jacobson, Junxiao Hu, Benjamin Ringel, Max A. Seibold, Craig P. Hersh, Katerina J. Kechris, Russell P. Bowler

**Affiliations:** 1 Department of Biostatistics and Informatics, Colorado School of Public Health, University of Colorado Denver, Aurora, Colorado, United States of America; 2 Channing Division of Network Medicine, Brigham and Women’s Hospital, Boston, MA, United States of America; 3 Department of Medicine, National Jewish Health, Denver, Colorado, United States of America; 4 Center for Genes, Environment and Health, Department of Pediatrics, National Jewish Health, Denver, Colorado, United States of America; 5 Division of Pulmonary Sciences and Critical Care Medicine, University of Colorado School of Medicine, Aurora, Colorado, United States of America; Lee Kong Chian School of Medicine, SINGAPORE

## Abstract

Chronic obstructive pulmonary disease (COPD) occurs typically in current or former smokers, but only a minority of people with smoking history develops the disease. Besides environmental factors, genetics is an important risk factor for COPD. However, the relationship between genetics, environment and phenotypes is not well understood. Sample sizes for genome-wide expression studies based on lung tissue have been small due to the invasive nature of sample collection. Increasing evidence for the systemic nature of the disease makes blood a good alternative source to study the disease, but there have also been few large-scale blood genomic studies in COPD. Due to the complexity and heterogeneity of COPD, examining groups of interacting genes may have more relevance than identifying individual genes. Therefore, we used Weighted Gene Co-expression Network Analysis to find groups of genes (modules) that are highly connected. However, module definitions may vary between individual data sets. To alleviate this problem, we used a consensus module definition based on two cohorts, COPDGene and ECLIPSE. We studied the relationship between the consensus modules and COPD phenotypes airflow obstruction and emphysema. We also used these consensus module definitions on an independent cohort (TESRA) and performed a meta analysis involving all data sets. We found several modules that are associated with COPD phenotypes, are enriched in functional categories and are overrepresented for cell-type specific genes. Of the 14 consensus modules, three were strongly associated with airflow obstruction (meta *p* ≤ 0.0002), and two had some association with emphysema (meta *p* ≤ 0.06); some associations were stronger in the case-control cohorts, and others in the cases-only subcohorts. Gene Ontology terms that were overrepresented included “immune response” and “defense response.” The cell types whose type-specific genes were overrepresented in modules (*p* < 0.05) included natural killer cells, dendritic cells, and neutrophils. Together, this is the largest investigation of gene blood expression in COPD with 469 cases in COPDGene, ECLIPSE and TESRA combined, with 6267 genes common to all data sets. Additional, we have 42 and 83 controls in COPDGene and ECLIPSE, respectively.

## Introduction

Chronic obstructive pulmonary disease (COPD) is the fourth leading cause of death in the US, and it is estimated that it will be the fifth most common cause of morbidity and third leading cause of mortality by 2020 (see [1] and references therein). Although COPD patients are typically current or former smokers, only a minority of current or former smokers have COPD. Besides environmental and behavioral factors, genetics is an important risk factor for the development of COPD. However, the disease is highly complex with widely heterogeneous phenotypes, and the relationship between genetics, environment, and phenotypes is not well understood.

To understand genetic influences on COPD, genome-wide expression studies, which comprehensively profile gene transcription, have been performed in lung tissue. However, the invasiveness of sample collection and resulting small sample sizes (n = 16-75) [2–7] of these studies have limited the ability to examine subphenotypes. Note that a recent publication [8] has a larger sample size of 111 severe COPD cases and 40 control smokers, but larger sample sizes remain rare for lung tissue-based studies. Blood is a less invasive way to collect biosamples. The rationale for using blood to study COPD is increasing evidence that COPD is a systemic disease [9], with several systemic biomarkers associated with exacerbation of COPD (see [2] and references therein). Several studies of blood from COPD subjects have suggested that there are genomic signatures associated with COPD (see [2, 10, 11] and references therein). These studies were limited in that they were also of small sample size, did not have replications, and tended to examine individual genes in isolation.

The focus of gene expression analysis has shifted in recent years from the detection of individually expressed genes to the detection of groups or modules of genes that act together. The rationale is that individual genes might have small effects on or insignificant associations with a phenotype, but that a group of interacting genes with individually small effects might have a more significant association. Moreover, such groups of genes or modules often come from biologically known networks, which makes the interpretation and reproducibility easier—particularly since individually highly ranked genes can be poorly annotated or are often not reproducible across studies [12, 13].

Finding clinically or biologically relevant groups or modules of genes that interact may give insight into biologic mechanisms that underlie the pathogenesis of COPD. One approach is based on the popular Weighted Gene Co-expression Network Analysis (WGCNA) [14, 15]. In WGCNA, genes belong to the same module (cluster) if, roughly speaking, they are highly connected as measured by the topological overlap (see [16, 17]). Module definitions naturally vary between data sets, and depending on the comparability of data sets and choice of methods and parameters within WGCNA, modules are not always preserved between data sets. The limitation to many publications that use WGCNA is that they do not validate their findings in independent cohorts.

To alleviate the variability of module definitions between data sets, we used two publicly available data sets from the COPDGene and ECLIPSE studies (see Materials and methods) to define consensus modules. This makes the module definitions more robust, making it more likely to find consistent modules of genes that are co-expressed. We studied these modules and their relationships with COPD outcomes for airflow obstruction, using spirometry to measure breathing capacity, and for emphysema, using computed tomography (CT) for lung imaging. We then used these consensus module definitions on an independent data set (TESRA) and performed a meta analysis involving all data sets. Next, we compared the module approach to published results of single gene-phenotype associations in [2]. Finally, we studied overrepresentation of cell-type specific genes in the modules.

Together, this is the largest investigation of gene blood expression in COPD with 469 cases in COPDGene, ECLIPSE and TESRA combined, with 6267 genes common to all data sets. Additional, we have 42 and 83 controls in COPDGene and ECLIPSE, respectively.

## Materials and methods

### Study populations

All subjects gave informed written consent and individual study protocols were reviewed and approved by their respective institutional review board (IRB):

COPDGene: This study was reviewed and approved to the IRB at National Jewish Health, Denver, Colorado.
ECLIPSE: The study is being conducted in accordance with the Declaration of Helsinki and good clinical practice guidelines, and has been approved by the relevant ethics and review boards at the participating centers.
TESRA: All subjects provided written informed consent. TESRA was approved by the Institutional Review Boards at all participating centers (see Additional file 1 in [18]).

The studies used include both subjects with COPD as well as smokers without evidence of COPD. Further details regarding subject recruitment and data collection can be found as follows: For COPDGene (Genetic Epidemiology of COPD) see [2, 19]; for ECLIPSE (Evaluation of COPD Longitudinally to Identify Predictive Surrogate Endpoints) see [20]; for TESRA (Treatment of Emphysema With a Gamma-Selective Retinoid Agonist) see [18, 21].

### Clinical data and definitions

COPD case and control definitions were made using forced vital capacity (FVC), which is the volume that can be blown out after full inspiration; forced expiratory volume in 1 second (FEV_1_); FEV_1_/FVC; and FEV_1_% (percent predicted), which is FEV_1_ divided by predicted FEV_1_ in a population of same gender, race and adjusted for age and height. Values after bronchodilator treatment were used. Cases of COPD have FEV_1_/FVC < 0.7. COPD severity is further defined by GOLD Stages I-IV, corresponding to mild (GOLD I) if FEV_1_% ≥ 80, moderate (GOLD II) if 50% ≤ FEV_1_% < 80%, severe (GOLD III) if 30% ≤ FEV_1_% < 50%, and very severe (GOLD IV) if FEV_1_% < 30% [22]. Preserved ratio impaired spirometry (PRISm) is defined as FEV_1_/FVC ≥ 0.7 and FEV_1_% < 80% [23]. We excluded PRISm subjects from the analyses.

Emphysema was assessed by low attenuation area (LAA) measured by percent voxel density below -950 or -910 Hounsfield Units (HU). The lower the voxel density, the easier the X-rays can penetrate the lung, indicating larger airspaces. Not all studies used the same threshold, therefore whenever -950HU data was available, as in the COPDGene and Eclipse studies, we used this rather than the -910HU threshold. For TESRA, only -910HU threshold was available, which could be compared with the -910HU threshold available for COPDGene, but not ECLIPSE. Exacerbations were defined as an acute worsening of respiratory symptoms characterized by cough, sputum, and shortness of breath associated with either antibiotic or corticosteroid use.

We considered the clinical variables FEV_1_%, FEV_1_/FVC, and emphysema. However, instead of studying associations of these clinical variables with the gene modules directly, we adjusted the clinical variables for covariates using linear and beta regression models and considered the resulting residuals as outcomes (see S1 Table); we still refer to these variables as FEV_1_%, FEV_1_/FVC, and emphysema, respectively. We also refer to FEV_1_% and FEV_1_/FVC as spirometry phenotypes and to emphysema as CT phenotype.

### Microarray data

#### Data pre-processing

**COPDGene and ECLIPSE** The microarray datasets are available through Gene Expression Omnibus (GEO). The GEO accession numbers are GSE42057 for COPDGene and GSE54837 for ECLIPSE. Gene expressions were measured using Affymetrix Human Genome U133 plus 2.0 Gene Array (Affymetrix, Santa Clara, CA). The data were processed using Bioconductor packages in R. Data were filtered for present/absent frequency (only probe sets with present calls in all samples were used) and normalized using the RMA algorithm (rma function in “affy” package [24]).

**TESRA**: Gene expression were measured using Affymetrix Human Genome U133 plus 2.0 Array. All 54,675 probes passing quality control were retained regardless of level of variance across samples. These data were background corrected and quantile normalized using the robust multiarray average (RMA) method via the R Bioconductor package affy [24]. Of the 270 original subjects (arrays), we studied the 248 subjects that self-identified as Caucasian and clustered with the Caucasian subjects based on genetic data (see [18] for details).

Note that all the above data sets come from the same platform, Affymetrix. We considered an additional data set from Lineagen [25]. However, this data set was produced using the Illumina Omni-Express Chip. In general, gene expression levels and their correlations are difficult to compare between different platforms. We thus did not include this resource in our analysis, but note the platform dependency as a limitation to our meta-analysis.

#### Mapping of probes to genes

For comparison purpose, probes were mapped to gene symbol for each dataset using “collapseRows” function provided in R package “WGCNA”, and genes with multiple probes were represented by the probe with the max arithmetic mean value [26]. The final expression file for each data set contains only the probe sets with associated genes; and the selected probe set were reassigned with the name of their corresponding gene symbol. Our analysis was based on the 6267 genes that were common to all data sets.

#### Consensus network construction and module definition using WGCNA

All datasets were analyzed using the R package “WGCNA” [14] among other standard R functions. We constructed consensus modules based on the two publicly available microarray data sets from COPDGene and ECLIPSE, and we applied the module definitions to the independent TESRA microarray data.

The network construction was based on the adjacency matrices of individual data sets. We used a signed network, where the adjacency matrix, *S* = (*S*_*ij*_), was of the form

$$S_{ij}={\left(\frac{1+\text{cor}\left(g_{i},g_{j}\right)}{2}\right)}^{\beta },$$

where cor(*g*_*i*_, *g*_*j*_) denotes the Pearson correlation between the expression profiles of gene *i* and *j*, respectively, and *β* is a user-defined parameter to suppress noisy small correlations. We based the choice of *β* on a scale free topology criterion of signed *R*^2^ ≥ 0.8 (see [17] for details on scale free topology). For COPDGene and ECLIPSE, we chose *β* = 13 and 21, respectively, resulting in signed *R*^2^ of 0.83 and 0.80, respectively.

Based on each adjacency matrix, the corresponding topological overlap matrix (TOM) was calculated (using the TOMsimilarity function in the WGCNA package). The consensus TOM was calculated component-wise as the minimum of the two individual TOM matrices. The individual TOM matrices were scaled before taking the minima to make them more comparable.

The individual and consensus TOMs were then used to define gene trees using geneTree = hclust(as.dist(1-TOM), method = “average”). The preliminary modules were then obtained using: cutreeHybrid(dendro = geneTree, pamStage = FALSE, minClusterSize = 30, deepSplit = 0, distM = 1-TOM). For each preliminary module, the eigengene was calculated, which were then clustered as before. At a height of 0.25 in the resulting dendrogram, subtrees below that threshold were merged to define new clusters. During the clustering visualization, one individual from COPDGene (24449A) was removed from the analysis, since this expression profile was very different from the remaining cases.

#### Eigengene analysis

For each of the final consensus modules, eigengenes were calculated for each of the data sets and correlated to phenotypes. These *p*-values were used to calculate a *Z*-score and meta *p*-value (for each module and phenotype) using a weighted version of Stouffer’s method: First, each *p*-value, *p*_*i*_, was transformed to a *Z*-score, *Z*_*i*_; a combined *Z*-score was then calculated as

$$\begin{array}{c} Z:=\frac{\sum_{i=1}^{k}w_{i}Z_{i}}{\sqrt{\sum_{i=1}^{k}w_{i}^{2}}}, \end{array}$$

where the weights *w*_*i*_ are the square-root transformed sample sizes of the individual data sets.

#### Module membership analysis

For each module and gene, we calculated the module membership kME, which is the correlation between the gene and the eigengene of the module [27]. For modules where the eigengenes were significantly associated with a clinical phenotype, it is of interest to examine whether specific genes in the modules are positively or negatively correlated with the phenotype. We restricted this analysis to the case-control control cohort of COPDGene and ECLIPSE. For each module, we rank the genes in each of the two cohorts according to kME and calculate the rank sum based on the two cohorts.

#### Gene-annotation enrichment analysis

We tested for overrepresentation (enrichment) of genes in each module in the Gene Ontology (GO) [28] categories *Biological Processes* and *Molecular Function*, using PANTHER (**P**rotein **AN**alysis **TH**rough **E**volutionary **R**elationships) [29]. Identifying such overrepresentations helps determining potential biological processes and molecular functions that the modules are involved in. The gene symbols were mapped to Gene ID and Protein ID prior to analysis; e.g. gene symbol AAK1 was mapped to Gene ID HGNC:19679 and Protein ID Q2M2I8 before starting the analysis. The background gene list was based on all 6267 genes studied. We filtered the list of GO terms reported by PANTHER to only include those that had at least 5 genes in the background gene list. For this reduced set of GO terms, we calculated FDR (false discovery rate) adjusted *p*-values according to Benjamini-Hochberg [30] using the R function p.adjust; FDR adjusted *p*-values were calculated on a per module basis across biological processes and molecular function categories combined. We then filtered the GO terms to only include the ones where fold enrichment was >1, since we were only interested in overrepresentation.

#### Comparison to previously published results

**Comparison to univariate analysis of the COPDGene cohort.** We compared our results with the univariate analysis of [2] in the COPDGene cohort, where the authors found individual genes whose relative abundance was associated with FEV_1_% and FEV_1_/FVC, based on linear model *t*-tests, correcting for covariates. They provided a list of genes with FDR adjusted *p*-values for each of the phenotypes. Those genes that had adjusted *p*-values smaller than 0.05 were considered significant. We used the intersection of these genes and the ones used in our module definition and performed overrepresentation tests of these genes and the ones in our modules using Fisher’s exact test.

**Comparison to previous ECLIPSE module-phenotype associations.** In [31], the authors applied WGCNA to a subset of the ECLIPSE cohort (discovery cohort), and studied the associations between the resulting modules and FEV_1_%. They tested the reproducibility on an independent sub-cohort of ECLIPSE (replication cohort). The three most significant modules in the discovery cohort were also the most significant ones in the replication cohort. We compared their module definitions with our consensus module definition on the genes that are common to their data and ours. We focused on the modules that were significant in [31] and in our study. We calculated the significance of the overlaps using Fisher’s exact test.

**Comparison to previous TESRA module-phenotype associations.** Similar to the comparison with ECLIPSE, we compared our results to the ones in [18], where the authors applied WGCNA to the TESRA cohort. A notable difference to our approach and the one in [31] is that the authors in [18] used probes rather than genes for their module definition. We thus first mapped their probes to genes the same way it has been done for the TESRA cohort in the current study. We then only considered genes that were common to this mapping and our consensus definition. Often, probes from different modules mapped to the same gene. We thus omitted genes that had multiple probes from different modules mapped to them. We also considered an alternative approach as follows. For a probe set that maps to a particular gene, and for which we have several module assignments, we used a “majority assignment,” in which we reassigned all probes of that set to the module with the most probes from that set. If there was a tie for the most probes in a module, we omitted the corresponding gene from comparison. We calculated the significance of the overlaps using Fisher’s exact test.

#### Module—Cell type relationship

We sought to identify gene expression signatures that exclusively identify immune cell-types. First, we aggregated flow sorted immune cell microarray data (111 samples) from Gene Expression Omnibus (GEO: GSE3982 and GSE22886). We identified 11 groups of distinct immune cell types consisting of eosinophils (eos), basophils/mast cells (mast_baso), dendritic cells (dendr), neutrophils (neut), b-cells, t-cells, NK-cells, t-helper cells (thelp), monocyte Lipopolysaccharides (LPS) day 0 stimulation, monocyte LPS day 1 stimulation, monocyte LPS day 7 stimulation (mono_d0, mono_d1, mono_d7, respectively). Differential expression analysis (limma) was performed between each cell type and all other cell types. Genes were selected based upon log-fold changes greater than 2 and an adjusted *p*-value less than 0.01. Using the significantly up-regulated genes, a random forest prediction model (R packages: caret, randomForest) was used to identify the most important subset for predicting each cell type from all other cell types. Genes with an importance measure greater than 0 were chosen for our final set of immune cell gene expression signatures. We tested for overrepresentation of these cell type signature genes in the consensus modules using Fisher’s exact test.

## Results

### Demographics

For COPD cases, the three cohorts are almost entirely Non-Hispanic White, and similar with respect to age, sex, BMI and ATS pack years (Table 1). However, the COPDGene cohort contains current smokers, while the other cohorts do not. The spirometry phenotypes are similar across the three cohorts. The GOLD stages for COPDGene are slightly higher than those of ECLIPSE, ranging from 1 to 4, while the TESRA cohort has mostly GOLD 2 and 3 stages. The controls in COPDGene and ECLIPSE are again almost all Non-Hispanic White, with similar BMI and age, although ECLIPSE has more males. Compared to the cases, the controls have better spirometry and CT phenotypes by definition. Furthermore, for both cohorts, controls have more females and are slightly younger. Note that TESRA does not have controls.

**Table 1:** Demographics.

	COPDGene cases	ECLIPSE cases	TESRA cases	COPDGene control	ECLIPSE control
n	83	138	248	42	83
Age (mean (sd))	64.70 (8.28)	64.92 (5.35)	66.4 (7.9)	60.42 (9.07)	61.29 (8.46)
Sex (number (%) males)	49 (59.04)	93 (67.4)	170 (68.6)	22 (52.38)	53 (63.86)
BMI (mean (sd))	27.77 (6.07)	26.93 (5.27)	26 (4.7)	27.98 (5.00)	28.02 (3.89)
Ethnicity/Race					
White	82 (98.80)	137 (99.28)	248 (100)	42 (100)	81 (97.59)
Black	1 (1.20)	0 (0)	0 (0)	0 (0)	2 (2.41)
Other	0 (0)	1 (0.72)	0 (0)	0 (0)	0 (0)
Current smoker (%)	17 (20.48)	0 (0)	0 (0)	13 (30.95)	0 (0)
Pack Years (mean (sd))	49.58 (24.64)	47.14 (29.33)	47 (24.3)	44.47 (28.49)	29.96 (26.97)
Percent Emph; -950 HU (mean (sd))^†^	13.58 (11.12)	18.59 (11.95)	NA	1.36 (1.51)	4.31 (3.79)
Percent Emph; -910 HU (mean (sd))^†^	39.80 (18.26)	NA	41.65 (16.2)	20.86 (11.74)	NA
FEV_1_% (mean (sd))	50.62 (21.13)	50.31 (16.68)	48.8 (9.3)	97.87 (13.80)	110.29 (14.58)
FEV_1_/FVC (mean (sd))	0.49 (0.14)	0.44 (0.12)	0.43 (0.09)	0.77 (0.04)	0.80 (0.06)
GOLD stage^††^ (%)					
GOLD 0	0 (0)	0 (0)	0 (0)	42 (100)	83 (100)
GOLD 1	6 (7.23)	3 (2.17)	0 (0)	0 (0)	0 (0)
GOLD 2	35 (42.17)	67 (48.55)	105 (42.3)	0 (0)	0 (0)
GOLD 3	25 (30.12)	55 (39.85)	139 (56.0)	0 (0)	0 (0)
GOLD 4	17 (20.48)	13 (9.42)	4 (1.6)	0 (0)	0 (0)
Exacerbation Freq. (mean (sd))	0.75 (1.16)	0.91 (1.57)	0.60 (0.65)	0.29 (0.86)	0 (0)

^†^ Seven and nine cases in COPDGene and ECLIPSE, respectively, did not have emphysema data. In the controls of ECLIPSE, 13 individuals had no emphysema data.

^††^ Cases of COPD all have FEV_1_/FVC < 0.7, and are further classified in GOLD Stages 1-4, corresponding to mild (FEV_1_% ≥80), moderate (50% ≤ FEV_1_% < 80%), severe (30% ≤ FEV_1_% <50%), and very severe (FEV_1_% < 30%) COPD [22].

### Consensus modules

**Fig 1:**
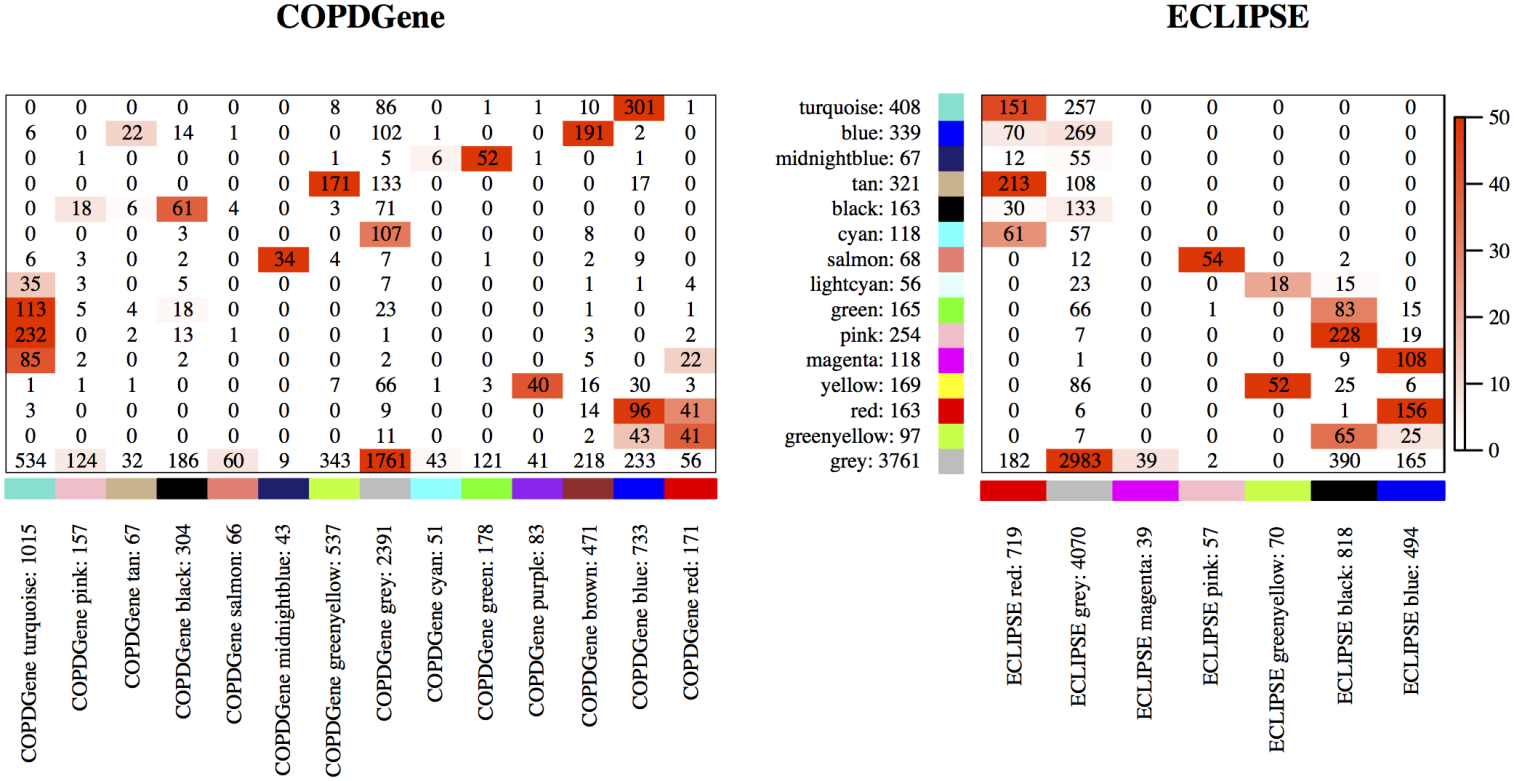
Correspondence of individual and consensus modules. Left (right, respectively) panel shows the correspondence between consensus modules and COPDGene (ECLIPSE, respectively) modules. The row labels give the color (label) of the consensus modules and the number of genes in the module. The columns represent in an analogous way the modules for COPDGene and ECLIPSE. The number in each cell gives the number of genes common to the modules in the corresponding row and column; the heatmap colors represent the −log_10_ transformed *p*-values (truncated at 50), which are based on Fisher’s exact test based on the hypergeometric distribution. Note that the module colors have no meaning, they simply represent consensus modules or modules in the individual data sets.

Gene co-expression modules were constructed separately for COPDGene and ECLIPSE, and then using a consensus approach (see Materials and methods). Most of the COPDGene modules significantly overlapped with only one consensus module (Fig 1). For ECLIPSE modules, there were more consensus module counterparts—this was partly a result of the lower number of ECLIPSE modules. The WGCNA method returns generally a module labeled “grey”, which contains genes that are not clustered with other co-expressed genes into modules—only non-grey modules represent co-expression modules. The grey module was the largest consensus module with 3761 genes and had strong overlap with the corresponding individual COPDGene and ECLIPSE grey modules. Besides the grey module, 14 consensus, 13 COPDGene, and 6 ECLIPSE modules were constructed. The largest non-grey module in COPDGene, labeled turquoise (1015 genes), had a significant overlap with several consensus modules. Note that the largest consensus module contained 408 genes. In our data, consensus module construction resulted in smaller modules as compared to constructions based on individual data sets. The gene module memberships are listed in S1 File.

### Module—Trait relationship

Using the cases-based consensus module definitions, we correlated phenotypes to module eigengenes (eigengene analysis) based on cases only or, where available, on cases and controls. The rationale for the latter is that eigengenes based on cases and controls represent a more diverse cohort.

**Fig 2:**
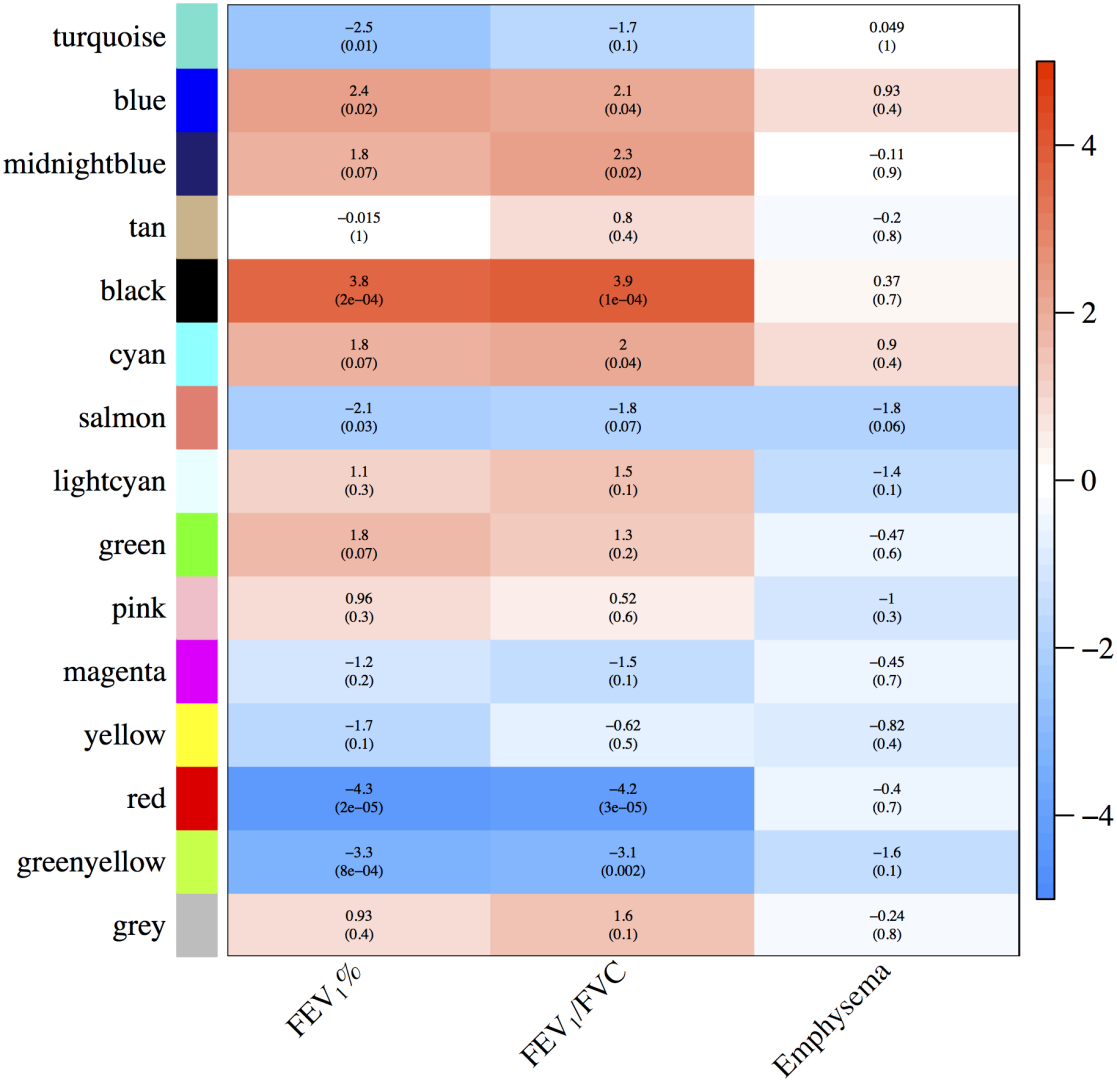
Consensus module−trait relationships across COPDGene and ECLIPSE for cases and controls. Meta-analysis *Z*-scores and *p*-values were based on cases and controls from COPDGene and ECLIPSE. The sign and magnitude of the *Z*-score give information about the overall direction and magnitude of association.

For COPDGene and ECLIPSE, we had control subjects defined by normal levels of spirometry. For these case-control cohorts, we performed eigengene analyses (S1 and S2 Figs), followed by a meta-analysis (Fig 2). Based on the meta-analysis, the most significant modules were black for FEV_1_% (*p* = 0.0002) and FEV_1_/FVC (*p* = 0.0001), red for FEV_1_% (*p* = 0.00002) and FEV_1_/FVC (*p* = 0.00003) and greenyellow for FEV_1_% (*p* = 0.0008) and FEV_1_/FVC (*p* = 0.002). The best candidate module for correlation with emphysema was the salmon module (*p* = 0.06). We saw good consistency between the two cohorts for these modules (S1 and S2 Figs).

**Fig 3:**
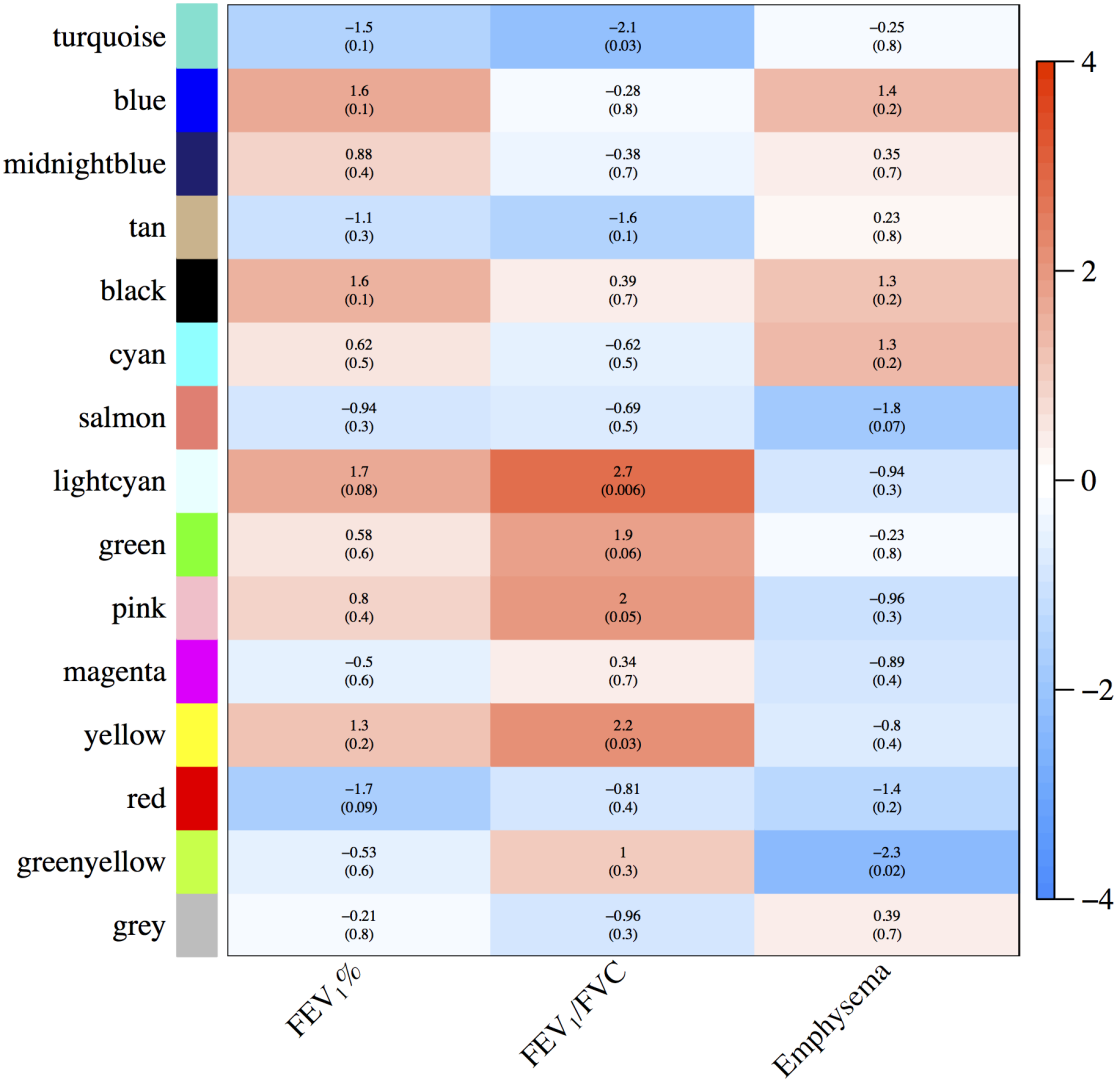
Consensus module-trait relationships across COPDGene, ECLIPSE, and TESRA for cases only. *Z*-scores and meta *p*-values for FEV_1_% and FEV_1_/FVC were based on COPDGene, ECLIPSE, and TESRA cases. The meta *p*-value for emphysema was based on COPDGene and ECLIPSE only, since the definition of emphysema in TESRA differed (see Materials and methods). As in Fig 2, the relationship information is given in terms of *Z*-scores rather than correlations.

For TESRA, we only had cases, and so we performed eigengene analyses using only cases from COPDGene, ECLIPSE, and TESRA (S3–S5 Figs), followed by a meta-analysis (Fig 3). Based on the latter, the most significant associations were between module eigengenes and FEV_1_/FVC, and the most significant modules for this trait were labeled lightcyan (*p* = 0.006), turquoise (*p* = 0.03), and yellow (*p* = 0.03). For correlation with emphysema, the most significant modules were greenyellow (*p* = 0.02) and salmon (*p* = 0.07). The best candidate module for correlation with FEV_1_% was lightcyan (*p* = 0.08).

Although the strength of correlations between individual genes and phenotypes or eigengenes varied between the cohorts, the genes with the highest module membership, based on the sum of ranks of kME (highest correlation with eigengene), tended to have the same gene-phenotype correlation direction. In particular, for the black, salmon, red, and greenyellow modules, we saw this consistency of top kME gene-phenotype relationships as well as moderate consistency in the kME ranking between the two cohorts (Table 2; see also (S2 File) for a complete list of module memberships and gene-phenotype correlations). Note that eigengenes of the black, red, and greenyellow modules were associated with the spirometry phenotypes, while the salmon module had the strongest (negative) association with emphysema among all modules, based on the COPDGene and ECLIPSE case-control cohorts. In the Salmon model we saw that out of the top 10 kME genes, only one was significantly associated with emphysema at the *α* = 0.05 level, namely XAF1 (*p* = 0.037).

**Table 2:** Top 10 module membership genes. The module membership of a gene (kME) is the correlation between the gene and the eigengene of the module. For the black, salmon, red and greenyellow modules, we list the top 10 kME genes based on the sum of ranks in the COPDGene and ECLIPSE cohort. The ranks were based on kME. For COPDGene and ECLIPSE, the correlation direction between the gene and the phenotypes are represented by “+” and “-”, if the correlation is significant at *α* = 0.05.

Module	Genes	Rank			COPDGene			ECLIPSE		
		COPDGene	ECLIPSE	Sum	FEV_1_%	FEV_1_/FVC	Emph.	FEV_1_%	FEV_1_/FVC	Emph.
red 163 genes	FCGRT	5	4	9	-	-		-	-	
	TALDO1	8	10	18	-	-				
	NFAM1	11	15	26	-	-				
	TYROBP	2	28	30	-	-		-	-	
	PFKFB4	18	14	32	-	-		-	-	
	ZNF467	21	11	32	-	-				
	FES	12	21	33	-	-		-	-	
	CFP	3	32	35	-	-		-	-	
	LTBR	6	29	35	-	-		-	-	
	TKT	20	17	37	-	-		-	-	
black 163 genes	KLF12	2	1	3	+			+	+	
	PLEKHA1	9	2	11	+	+		+	+	
	OXCT1	10	7	17	+			+	+	
	SLAIN1	3	20	23	+	+		+	+	
	PSIP1	23	3	26	+	+		+	+	
	RASGRP1	32	4	36	+	+		+	+	
	CDC42SE2	27	11	38	+	+		+	+	
	DDHD2	36	12	48	+	+		+	+	
	ITK	25	23	48	+	+		+	+	
	TC2N	13	37	50						
greenyellow 97 genes	EIF4EBP1	7	1	8	-	-				
	PYCARD	2	6	8	-	-		-	-	
	TMEM205	8	2	10	-	-				
	FAM127A	10	5	15	-					
	AGTRAP	4	12	16	-	-		-	-	
	AP2S1	13	3	16		-				
	KIAA0930	5	15	20	-	-		-	-	
	NRM	17	7	24	-					
	TSPO	3	22	25	-			-		
	ZNF385A	9	16	25	-	-				
salmon 68 genes	IFI35	3	2	5				-	-	
	XAF1	5	1	6						-
	IFIT3	7	3	10				-	-	
	HERC5	4	7	11						
	RSAD2	2	9	11				-	-	
	MX1	13	5	18						
	IFI6	6	13	19				-	-	
	UBE2L6	1	18	19						
	IFI44L	9	11	20						
	OASL	14	6	20				-	-	

### Gene-annotation enrichment analysis

Based on the module-trait relationship analysis of the case-control cohorts COPDGene and ECLIPSE, the red, black, greenyellow, salmon modules were most significantly associated with the phenotypes. These phenotype-related modules were overrepresented in GO categories of biological processes (Table 3 and S3 File). Example of enriched GO terms include “immune response,” “defense response,” and signaling related terms in the red module; “regulation of natural killer cell activation” and “positive regulation of natural killer cell mediated cytotoxicity” as well as terms related to cell cycle and DNA checkpoints in the black module; “immune response” and several interferon related terms in the salmon module; “multicellular organismal response to stress” and energy related terms in the greenyellow module. Other modules not as strongly associated with the phenotypes considered might be of independent interest for other smoking related diseases. These modules were also overrepresented in GO terms of molecular function (S2 Table and S3 File).

**Table 3:** Panther results for biological processes. For each of the salmon, red, greenyellow, and black module, the top 10 enriched biological processes GO terms are presented. The reference list consists of the 6322 genes that were mapped by PANTHER and were common to all data sets and used for module construction. The # Hits denotes the number of genes in the module that are in the GO term. Fold Enrichment gives the overrepresentation. The *p*-values were calculated by the PANTHER software based on the binomial distribution. They were adjusted based on the Benjamini-Hochberg procedure on a per module basis, considering all tests for biological processes and molecular function (see S2 Table for molecular function GO term results).

Module	Top Biological Processes	Ref. list 6322 genes	# Hits	Fold Enrich.	p-value	p.adjust
red 168 genes	immune response (GO:0006955)	441	33	2.82	6.1E-08	3.6E-05
	defense response (GO:0006952)	454	31	2.57	1.2E-06	3.4E-04
	actin polymerization or depolymerization (GO:0008154)	21	7	12.54	1.9E-06	3.6E-04
	single organism signaling (GO:0044700)	1613	70	1.63	3.6E-06	3.6E-04
	signaling (GO:0023052)	1615	70	1.63	3.8E-06	3.6E-04
	signal transduction (GO:0007165)	1555	68	1.65	4.2E-06	3.6E-04
	actin filament organization (GO:0007015)	75	11	5.52	6.4E-06	4.7E-04
	immune system process (GO:0002376)	864	44	1.92	1.3E-05	7.7E-04
	actin filament polymerization (GO:0030041)	11	5	17.1	1.3E-05	7.7E-04
	cell communication (GO:0007154)	1649	69	1.57	1.7E-05	9.0E-04
black 164 genes	mitotic cell cycle checkpoint (GO:0007093)	78	8	3.95	1.1E-03	2.6E-02
	regulation of natural killer cell activation (GO:0032814)	10	3	11.56	2.4E-03	3.6E-02
	mitotic DNA integrity checkpoint (GO:0044774)	54	6	4.28	3.0E-03	4.0E-02
	DNA integrity checkpoint (GO:0031570)	78	7	3.46	4.5E-03	4.3E-02
	cell cycle checkpoint (GO:0000075)	103	8	2.99	5.8E-03	4.3E-02
	negative regulation of mitotic cell cycle (GO:0045930)	107	8	2.88	7.2E-03	4.3E-02
	snoRNA localization (GO:0048254)	5	2	15.42	7.7E-03	4.3E-02
	pos. regul. of lymphocyte mediated immunity (GO:0002708)	31	4	4.97	9.0E-03	4.3E-02
	regulation of interleukin-10 production (GO:0032653)	17	3	6.8	1.0E-02	4.3E-02
	neural tube patterning (GO:0021532)	6	2	12.85	1.1E-02	4.3E-02
greenyellow 100 genes	multicellular organismal response to stress (GO:0033555)	17	4	14.88	1.7E-04	4.5E-03
	hexose metabolic process (GO:0019318)	77	7	5.75	2.4E-04	6.0E-03
	monosaccharide metabolic process (GO:0005996)	81	7	5.46	3.2E-04	6.6E-03
	hexose catabolic process (GO:0019320)	21	4	12.04	3.7E-04	6.6E-03
	glutathione derivative biosynthetic process (GO:1901687)	9	3	21.07	4.2E-04	6.6E-03
	glutathione derivative metabolic process (GO:1901685)	9	3	21.07	4.2E-04	6.6E-03
	monosaccharide catabolic process (GO:0046365)	22	4	11.49	4.4E-04	6.6E-03
	glucose metabolic process (GO:0006006)	62	6	6.12	4.8E-04	6.8E-03
	regulation of endopeptidase activity (GO:0052548)	146	9	3.9	5.4E-04	7.3E-03
	regulation of peptidase activity (GO:0052547)	151	9	3.77	6.8E-04	7.7E-03
salmon 68 genes	type I interferon signaling pathway (GO:0060337)	38	19	46.49	1.6E-26	2.3E-24
	cellular response to type I interferon (GO:0071357)	38	19	46.49	1.6E-26	2.3E-24
	response to type I interferon (GO:0034340)	42	19	42.06	1.0E-25	9.9E-24
	cytokine-mediated signaling pathway (GO:0019221)	195	27	12.87	3.2E-23	2.3E-21
	innate immune response (GO:0045087)	239	28	10.89	3.4E-22	1.9E-20
	defense response to virus (GO:0051607)	80	20	23.24	5.1E-22	2.4E-20
	response to virus (GO:0009615)	125	22	16.36	5.4E-21	2.2E-19
	immune response (GO:0006955)	441	33	6.96	1.6E-20	5.9E-19
	response to cytokine (GO:0034097)	316	29	8.53	3.7E-20	1.2E-18
	cellular response to cytokine stimulus (GO:0071345)	264	27	9.51	7.3E-20	2.1E-18

### Comparison to previously published results

#### Comparison to univariate analysis of the COPDGene cohort

Of the 6267 genes used in the module definition, 523 (or 8.34%) were previously found to be significant in a univariate analysis for FEV_1_% and FEV_1_/FVC in the COPDGene cohort [2]. Those genes were overrepresented in two modules: 25 of the 163 red module genes (or 15.34%) were significant in the univariate list (Fisher’s exact *p* = 0.002 for overrepresentation), and 116 of the 408 turquoise module genes (or 28.43%) were significant in the univariate list (Fisher’s exact *p* < 2.2 × 10^−16^).

#### Comparison to previous ECLIPSE module-phenotype associations

In the ECLIPSE study [31], module associations with FEV_1_% were considered, with the most significant ones labeled in their study as yellow, green and brown, in both their discovery and replication cohorts. While we calculated the overlap between all modules (S6 Fig), we focused on the significant consensus modules for FEV_1_% discovered in our work (black, red and greenyellow). Our black module significantly overlaps with ECLIPSE’s blue (*p* = 2.4E-13) and brown (*p* = 2.9E-06), based on Fisher’s exact test. Note that ECLIPSE’s blue module shows some evidence of association with FEV_1_% as well (FDR corrected *p* = 5.97E-02 in discovery cohort). Our red module overlaps with green (*p* = 4.9E-06) and yellow (*p* = 7.0E-84) in ECLIPSE, and our greenyellow module overlaps with magenta (*p* = 4.1E-04), red (*p* = 3.0E-07), and yellow (*p* = 4.4E-11). Note that ECLIPSE’s magenta and red modules show some evidence of association with FEV_1_% as well (FDR corrected *p* = 9.31E-02 and *p* = 1.52E-01 for the magenta and red modules, respectively, in discovery cohort).

#### Comparison to previous TESRA module-phenotype associations

In the TESRA study [18], module associations with FEV_1_/FVC were considered, with the most significant ones labeled in their study as darkturquoise, purple, saddlebrown, and cyan, with FDR adjusted p-values 0.19, 0.19, 0.19, and 0.2, respectively. Note that they did not consider associations with FEV_1_% or emphysema. We thus considered the overlap between the above mentioned TESRA modules and the three consensus modules with the strongest association with FEV_1_/FVC in our work, which are the same as for FEV_1_%—black, red and greenyellow. TESRA’s modules were defined on the probe level rather than gene level, and we used the two approaches discussed in Materials and methods. The significance of the overlap were quite similar between methods, so we only present the results from the first approach (S7 Fig). There was no overlap between any of our three significant modules and TESRA’s darkturquoise, purple, saddlebrown, and cyan modules.

### Module—Cell type relationship

For some modules, there was overrepresentation of cell type specific genes, based on a significance level of *α* = 0.05 using Fisher’s exact test (Table 4 and S3 Table). Some cell types were overrepresented in only one non-grey module (monocyte Lipopolysaccharides day 1 and day 7 stimulation, dendritic cells), while others were overrepresented in two or more modules (monocyte Lipopolysaccharides day 0 stimulation, NK-cells, neutrophils). T-cells, b-cells, eosinophils, basophils/mast cells, and t-helper cells were not overrepresented in any of the colored modules; however, t-cells and t-helper cells were overrepresented in the grey module. On the other hand, for each module, except for the turquoise and grey modules, there was at most one cell type that is overrepresented.

**Table 4:** Overrepresentation of cell-type specific genes in modules. Only overrepresentations significant at level *α* = 0.05 are displayed for readability. For a complete table of *p*-values, see S3 Table. The 11 groups of distinct immune cell types considered consist of eosinophils (eos), basophils/mast cells (mast_baso), dendritic cells (dendr), neutrophils (neut), b-cells, t-cells, NK-cells, t-helper cells (thelp), monocyte Lipopolysaccharides (LPS) day 0 stimulation, monocyte LPS day 1 stimulation, monocyte LPS day 7 stimulation (mono_d0, mono_d1, mono_d7, respectively).

	mono_d0	mono_d1	mono_d7	NK-cell	t-cell	b-cell	neut	dendr	eos	mast_baso	thelp
turquoise	6.3E-03						3.0E-02				
blue		1.5E-02									
midnightblue											
tan			2.4E-03								
black				9.1E-03							
cyan											
salmon								2.1E-02			
lightcyan											
green				1.7E-02							
pink											
magenta							1.8E-02				
yellow											
red							7.9E-06				
greenyellow	1.3E-03										
grey					1.8E-02						2.7E-05

## Discussion

COPD is a highly complex disease with widely heterogeneous phenotypes, and the relationship between genetics, environment, and phenotypes is not well understood. Previous work has suggested that COPD is a systemic disease [9] and that there may be genomic signatures in the blood [2]. We focused on the construction and analysis of consensus modules in blood to find groups of genes that as a group have significant disease associations and biological significance. This analysis included the largest collection of blood gene expression profiles in COPD to-date with nearly 600 subjects across three cohorts. The consensus modules were defined based on gene co-expression (WGCNA) in COPD cases from the COPDGene and ECLIPSE cohorts to give us stable disease related modules, which were then validated on an independent cohort (TESRA). From this work, we were able to uncover networks of co-expressed genes that show consistent phenotype associations across multiple cohorts.

The advantages of gene module analysis compared to a univariate analysis is that individual genes might have small effects on or insignificant associations with a phenotype, but that a group of interacting genes with individually small effects might have a more significant association. Moreover, such groups of genes often come from biologically known networks, which makes the interpretation and reproducibility easier—particularly since individually highly ranked genes can be poorly annotated or are often not reproducible across studies [12, 13]. Indeed, genes that were previously found to be associated with spirometry phenotypes [2] were overrepresented in specific modules (red and turquoise), which indicates that we can partially recover genes that can be found in a univariate analysis. However, not all genes present in the significant modules were found in the univariate analysis, and, vice versa, not all significant genes from the univariate analysis were found in significant modules. Thus, the module approach gives an alternative view to univariate gene-phenotype relationships, with potential benefits, particularly in Gene-annotation enrichment analysis. Testing each gene independently is prone to decreased power due to the large multiple testing burden resulting in false negatives. Moreover, in any univariate analysis false positives are also of major concern. We alleviated the problems of false positives and negatives through the module approach by reducing the multiple testing burden (recall that we tested module-phenotype associations rather than gene-phenotype associations), incorporating dependencies between genes, and using two cohorts for robust module definition. Moreover, we found several of our modules overrepresented for different known GO terms, which gave us an alternative view on overrepresentation as compared to univariate analyses. In the WGCNA module approach, we first grouped genes into modules, and then looked for overrepresentation of these module-genes. Overrepresentation was tested within each module as opposed to within all genes that are significant at an individual level.

When studying module-trait relationships, we first considered the COPDGene and ECLIPSE case-control cohorts, followed by an analysis based on the cases of all cohorts. While the associations in the cases-cohorts are not as strong as in the complete cohorts due to the smaller sample size and a narrower range of outcomes, our results show relatively consistent association among different independent cohorts (COPDGene, ECLIPSE, and TESRA) and in the sub-populations (cases only versus cases and controls). We focused on two of the primary COPD phenotypes, airflow obstruction (FEV_1_% and FEV_1_/FVC) and CT assessed emphysema.

The modules that were most significantly associated with airflow obstruction in the two case-control cohorts (COPDGene and ECLIPSE) were the red, greenyellow, and black modules. The genes in the modules that were most correlated with the eigengenes had also consistent associations with airflow obstruction: these genes in the red and greenyellow modules were negatively correlated, while the genes in the black module were positively correlated. The trends were of similar magnitude and direction for spirometry measurements, for which FEV_1_% and FEV_1_/FVC tend to be highly correlated. In these three modules, the results were consistent in the case-control cohorts from COPDGene and ECLIPSE, and nearly consistent when considering their cases-only subpopulations, albeit with less significant associations. Out of the three modules, the TESRA cohort was consistent with the other two cohorts only for FEV_1_% in the black module. A possible reason for this is that the network definition was based only on the COPDGene and ECLIPSE cohorts, and the three modules were selected based on the association of the modules in these case-control cohorts. This suggests that the network approach could benefit from including more cohorts in the module definition. This can be seen as strength of the WGCNA approach when considering multiple cohorts, but it is also a limitation when considering only one cohort, which is often the case when using WGCNA. Another likely reason for less consistency between TESRA and COPDGene/ECLIPSE is that we get more reliable associations between modules and a phenotype when considering the whole spectrum of the phenotype across cases and controls.

We compared our consensus modules that had significant association with FEV_1_% (black, red, and greenyellow), with modules defined previously for ECLIPSE [31] and TESRA [18]. There was strong overlap of our three significant modules with ECLIPSE’s three most significant modules with respect to this phenotype, as well as with other ECLIPSE modules that showed evidence of association with FEV_1_%. This is despite the fact that in [31] more modules were defined than in our analysis. This could be expected to a certain degree given that we also used ECLIPSE in the consensus module definition. However, it is reassuring that independent analysis approaches using WGCNA gave consistent results.

There was little overlap between the modules of interest of our study and the previously published TESRA modules [18]. This may be a result of not using TESRA in the consensus module definition, and TESRA’s module definitions being defined using probe sets which we then mapped to genes in the overlap analysis. It could well be that both approaches lead to different views on the data, that are not quite comparable.

Gene ontology enrichment analysis provided information on functional categories that may be represented by the modules. The red module was overrepresented in GO terms such as “immune response,” “defense response,” and “immune system process.” Moreover, cytokine related terms were overrepresented; in a previous study using the COPDGene a cohort, COPD patients had a diminished cytokine response as compared to controls [32].

An additional module we considered was the turquoise module, whose associations with the spirometry phenotypes were not as consistent and strong, except for a strong association in the COPDGene case-control cohort. However, the turquoise module was the largest one, it showed evidence of phenotype association, and it was overrepresented in the significant genes from the univariate analysis in [2], which made it worth examining. The most significant overrepresentation in the turquoise module was for homeostatic process, which is a more general GO term. However, there were also more specific GO terms involving lipids and sphingolipids in the turquoise module (e.g., lipid metabolic process, cellular lipid metabolic process, sphingolipid metabolic process). Sphingolipids have been implicated in the pathogenesis of COPD (see [33] and references therein). Finally, when comparing our results to the list of significant genes for FEV_1_% and FEV_1_/FVC from the univariate analysis in [2], both the red and turquoise module were overrepresented in this list.

For emphysema, in the case-control cohorts of COPDGene and ECLIPSE, the salmon module was the best candidate module (meta *p* = 0.06), which was overrepresented in several immune response GO terms, and in particular, cytokine and interferon related terms; both cytokine response and interferon gamma levels were associated with COPD outcomes [32]. Note that the dendritic cell-specific genes were overrepresented in this module, which further points to its immune role. In the cases-only cohorts, the greenyellow module was associated with emphysema (meta *p* = 0.02). In this module, sphingolipid terms were overrepresented; sphingolipids have been associated with emphysema [33].

While including genomic data from three cohorts was a strength of this study, there were challenges presented by some of the heterogeneity of the cohorts. For instance, the TESRA cohort had only COPD subjects. In these sub-cohorts, we saw less significant results than in the case-control cohorts, which is likely due to the smaller sample sizes and a narrower range of outcomes, yielding less power. However, there was good consistency between the cohorts with respect to module-phenotype relationships. Another challenge to our meta-analysis was the platform dependency. We considered an additional data set from Lineagen [25]. However, this data set was produced using the Illumina Omni-Express Chip while the other sets used Affymetrix. In general, gene expression levels and their correlations are difficult to compare between platforms. We thus did not include this resource in our analysis. Despite these limitations, the consensus module approach demonstrated that we can identify gene modules that are significantly associated with COPD phenotypes. Some of these modules were enriched with genomic signatures identified in univariate analyses of spirometry associated genes [2, 33], but also included novel genes that fit in similar GO categories. This indicates that a combination of cases-based consensus modules and case-control based eigengene analysis can consistently find biologically relevant groups of genes, and that this approach might be more robust than univariate analyses that often cannot be reproduced in independent cohorts. The modules might serve as biomarkers, and provide insight into new targets for therapy. In summary, since the consensus gene modules were replicated in multiple cohorts and are highly connected, they are stronger candidates (compared to genes discovered using single gene approaches in only one cohort) for testing as biomarkers in additional cohorts or cohorts with different ethnic background, or mechanistically in cell or tissue functional studies.

## Supporting information

S1 Fig Module-trait relationships in COPDGene cases and controls. Module-trait relationship using consensus module definition for COPDGene cases and controls data. Correlations were calculated between module eigengenes and phenotypes; *p*-values are provided in brackets.
(TIFF)

S2 Fig Module-trait relationships in ECLIPSE cases and controls. Module-trait relationship using consensus module definition for ECLIPSE cases and controls data. Correlations were calculated between module eigengenes and phenotypes; *p*-values are provided in brackets.
(TIFF)

S3 Fig Module-trait relationships in COPDGene cases. Module-trait relationship using consensus module definition for COPDGene cases data. Correlations were calculated between module eigengenes and phenotypes; *p*-values are provided in brackets.
(TIFF)

S4 Fig Module-trait relationships in ECLIPSE cases. Module-trait relationship using consensus module definition for ECLIPSE cases data. Correlations were calculated between module eigengenes and phenotypes; *p*-values are provided in brackets.
(TIFF)

S5 Fig Module-trait relationships in TESRA cases. Module-trait relationship using consensus module definition for TESRA data. Correlations were calculated between module eigengenes and phenotypes; *p*-values are provided in brackets.
(TIFF)

S6 Fig Overlap between consensus modules and ECLIPSE module definitions in [31]. This overlap analysis is performed similarly to the one in Fig 1. The row labels give the color (label) of the consensus modules and the number of genes in the module. The columns represent in an analogous way the modules for the ECLIPSE modules from [31] (recall that we filtered the genes as described in Materials and methods). The number in each cell gives the number of genes common to the modules in the corresponding row and column; the heatmap colors represent the −log_10_ transformed *p*-values (truncated at 50), which are based on Fisher’s exact test. Note that the module colors have no meaning, they simply represent consensus modules or ECLIPSE’s modules.
(TIFF)

S7 Fig Overlap between consensus modules and TESRA module definitions in [18]. This overlap analysis is performed analogously to the one in S6 Fig, but with the TESRA cohort instead of the ECLIPSE cohort. See also Materials and methods for a filtering of the genes.
(TIFF)

S1 File Module definitions. This file provides a list of all modules and their genes.
(CSV)

S2 File Module membership (kME) and gene-phenotype correlations. These files provide the results from correlating genes with phenotypes as well as the genes’ module memberships kME, i.e. eigengene (EG) correlation.
(ZIP)

S3 File Panther results. This Microsoft Excel file lists the results from the enrichment analysis using PANTHER. The file contains one sheet for each module, listing the GO terms along with enrichment statistics and p-values.
(XLSX)

S1 Table The outcomes for the data sets from the COPDGene, ECLIPSE, and TESRA studies are adjusted for covariates using linear and beta regression. (PDF)

S2 Table Panther results for molecular function. The structure of this table is the same as of Table 3. The difference is that we considered molecular function GO terms rather than biological processes terms.
(PDF)

S3 Table Module—Cell relationship. Overrepresentation of cell type specific genes in consensus modules was calculated using Fisher’s exact test. The 11 groups of distinct immune cell types considered consist of eosinophils (eos), basophils/mast cells (mast_baso), dendritic cells (dendr), neutrophils (neut), b-cells, t-cells, NK-cells, t-helper cells (thelp), monocyte Lipopolysaccharides (LPS) day 0 stimulation, monocyte LPS day 1 stimulation, monocyte LPS day 7 stimulation (mono_d0, mono_d1, mono_d7, respectively).
(PDF)
