# Supplementary figures and images for: Meta-analysis of peripheral blood gene expression modules for COPD phenotypes
Source: PLoS One. 2017 Oct 9;12(10):e0185682. doi: 10.1371/journal.pone.0185682 (PMC5633174; doi:10.1371/journal.pone.0185682)

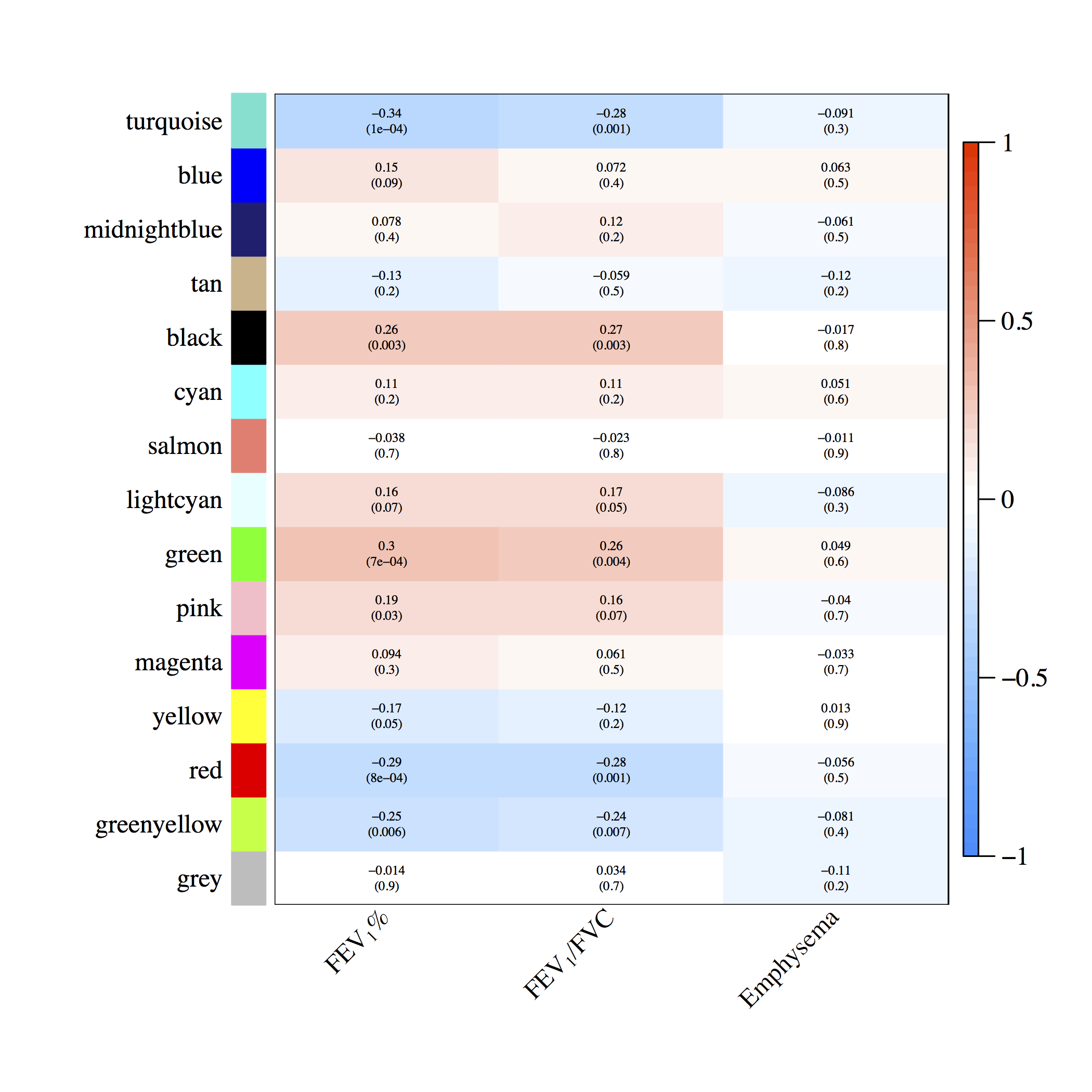

Supplement: S1 Fig — Module-trait relationship using consensus module definition for COPDGene cases and controls data. Correlations were calculated between module eigengenes and phenotypes; p-values are provided in brackets. (TIFF) [file pone.0185682.s001.tiff]

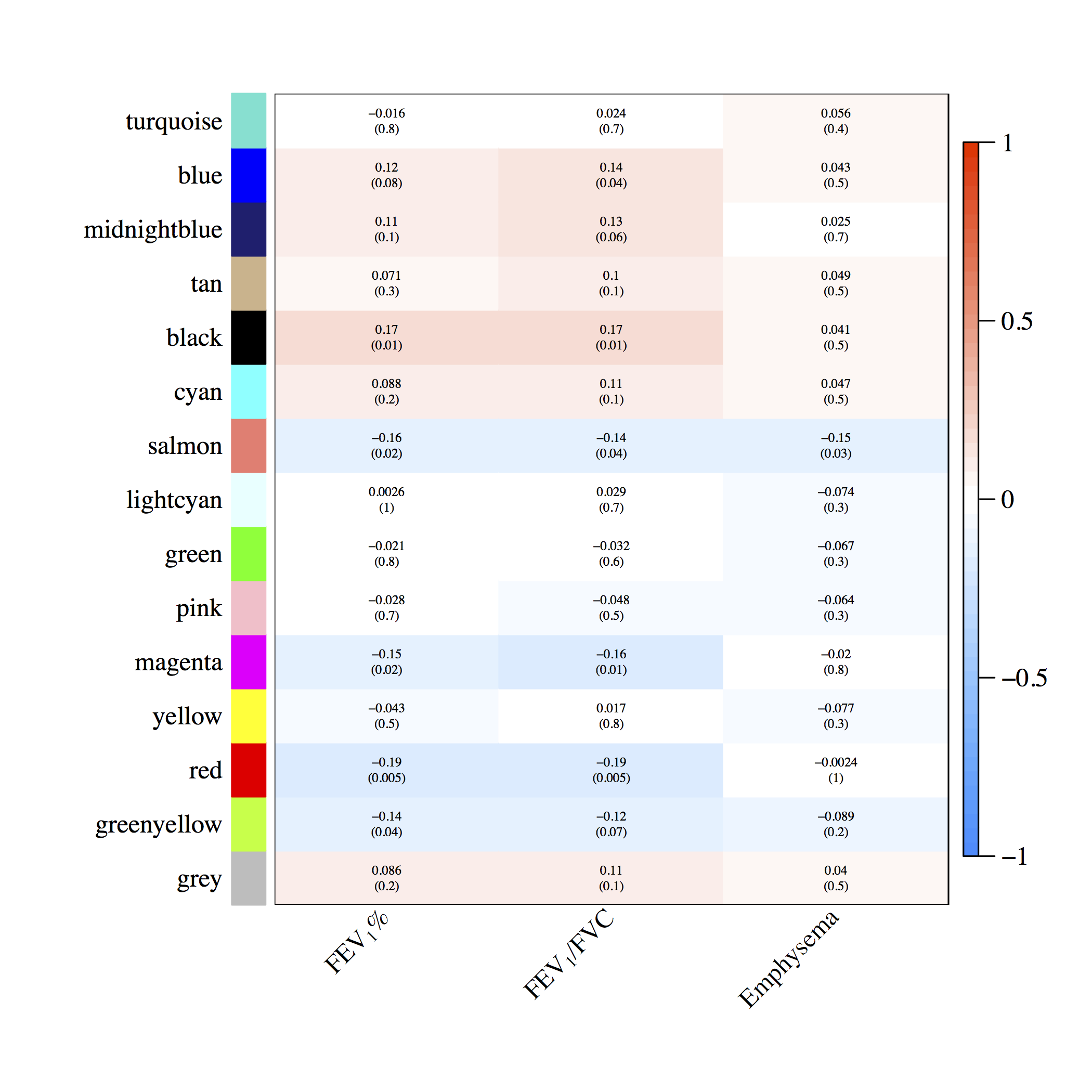

Supplement: S2 Fig — Module-trait relationship using consensus module definition for ECLIPSE cases and controls data. Correlations were calculated between module eigengenes and phenotypes; p-values are provided in brackets. (TIFF) [file pone.0185682.s002.tiff]

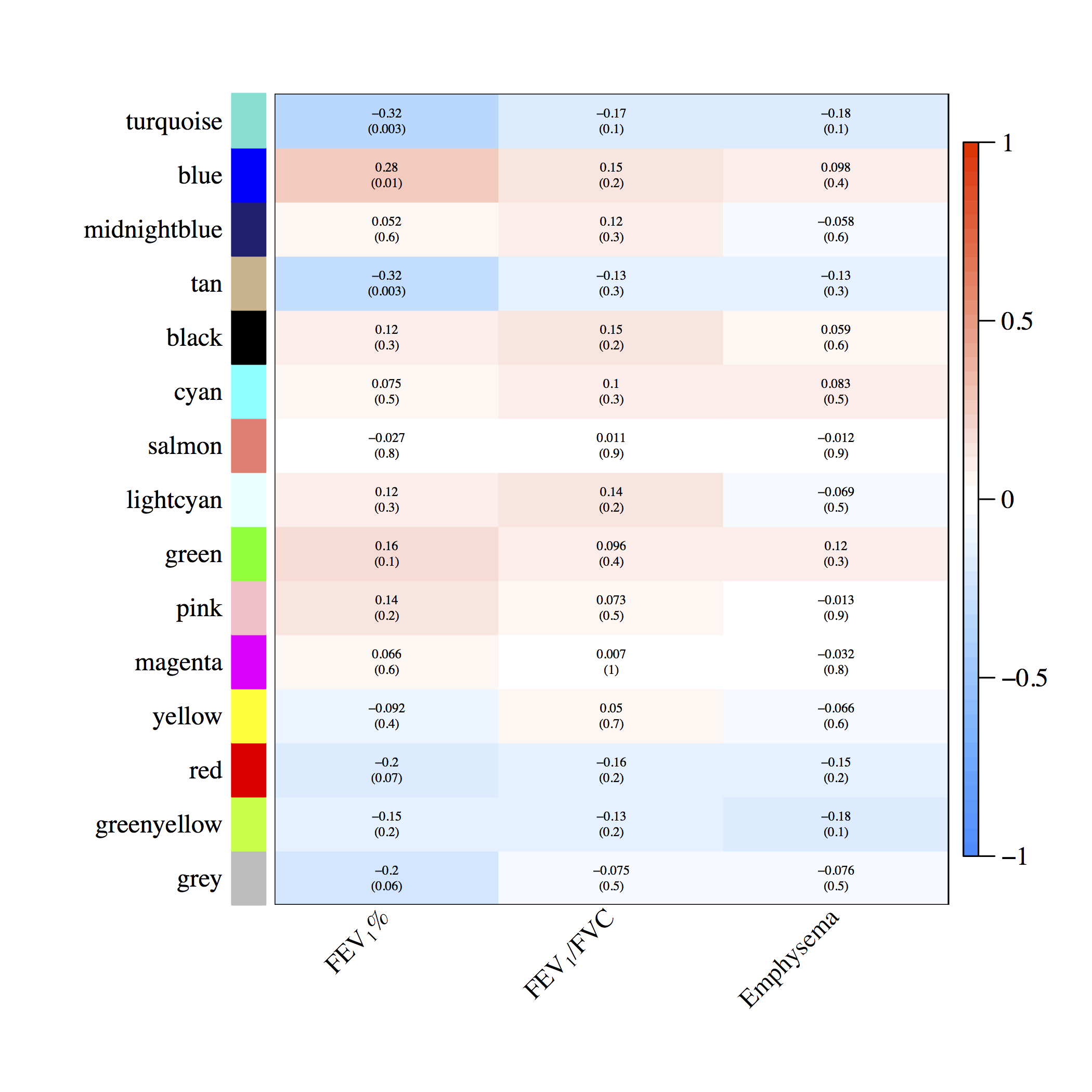

Supplement: S3 Fig — Module-trait relationship using consensus module definition for COPDGene cases data. Correlations were calculated between module eigengenes and phenotypes; p-values are provided in brackets. (TIFF) [file pone.0185682.s003.tiff]

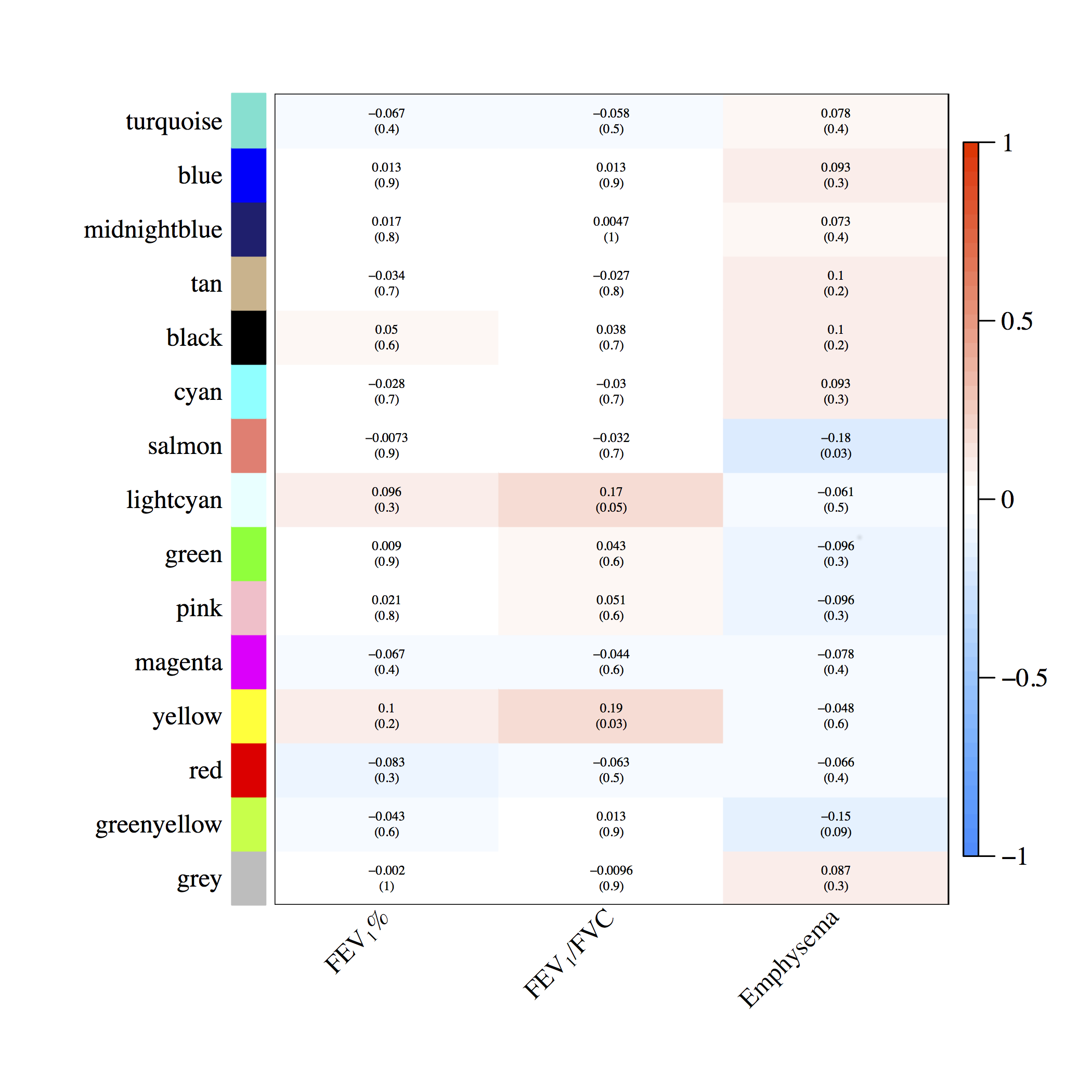

Supplement: S4 Fig — Module-trait relationship using consensus module definition for ECLIPSE cases data. Correlations were calculated between module eigengenes and phenotypes; p-values are provided in brackets. (TIFF) [file pone.0185682.s004.tiff]

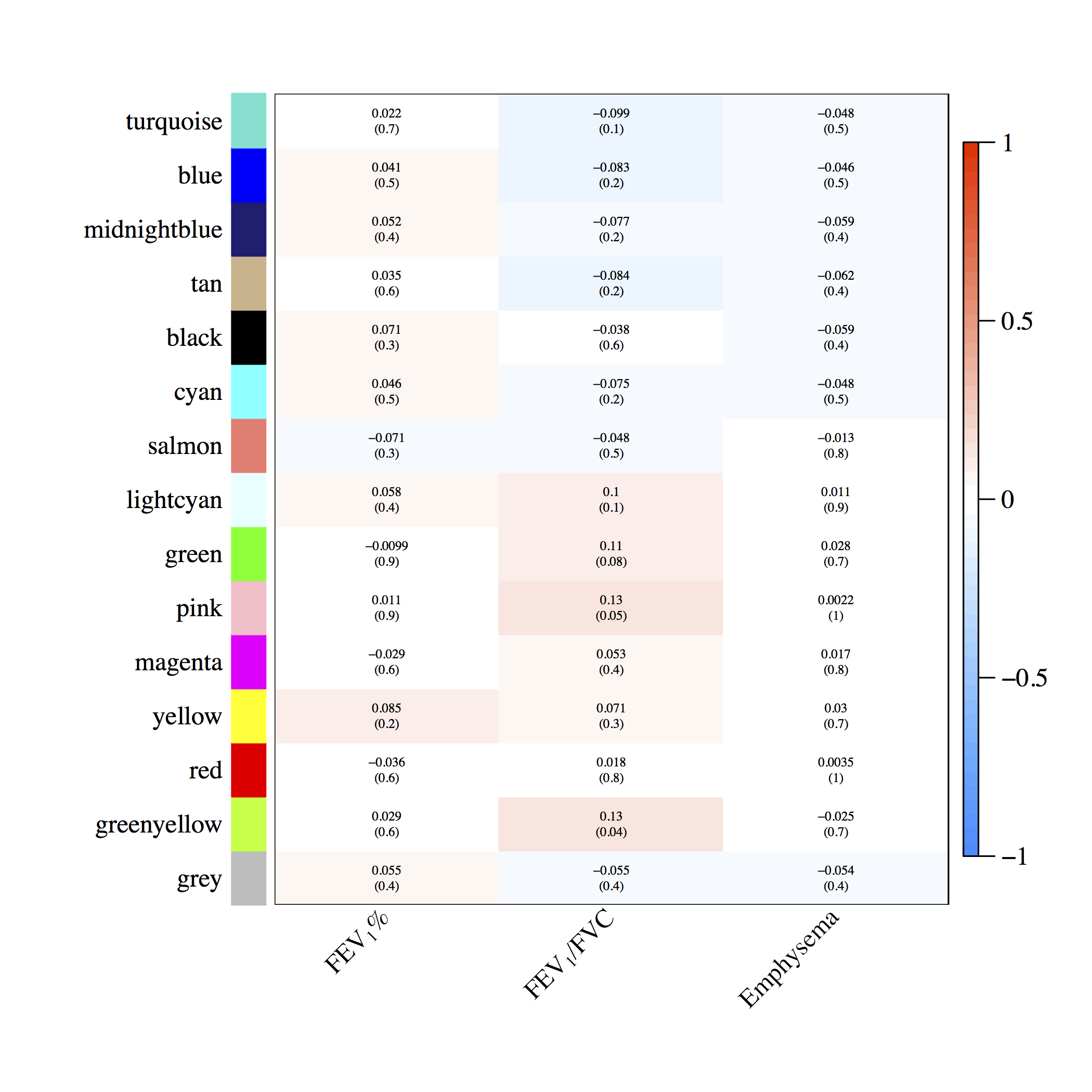

Supplement: S5 Fig — Module-trait relationship using consensus module definition for TESRA data. Correlations were calculated between module eigengenes and phenotypes; p-values are provided in brackets. (TIFF) [file pone.0185682.s005.tiff]

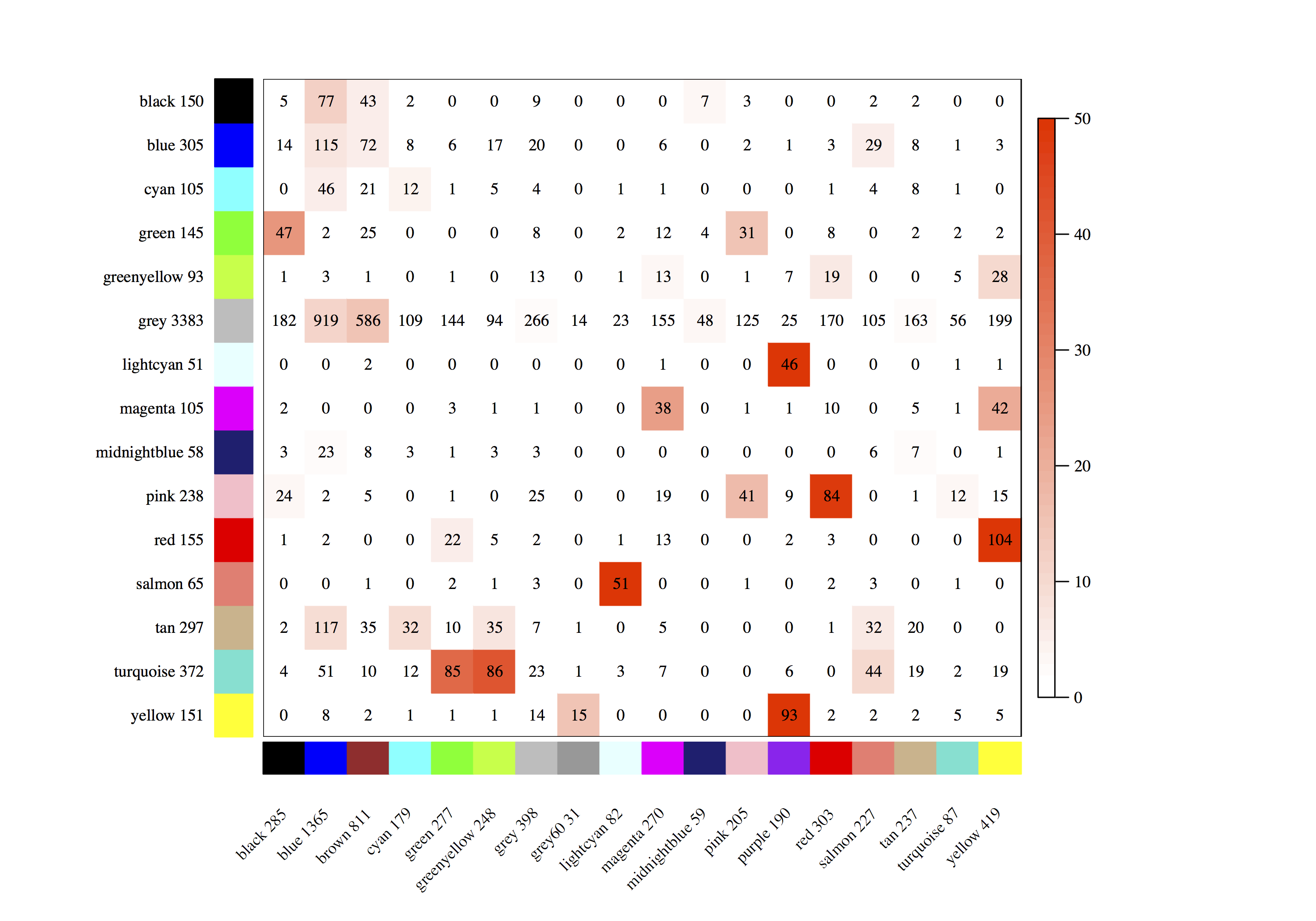

Supplement: S6 Fig — This overlap analysis is performed similarly to the one in Fig 1. The row labels give the color (label) of the consensus modules and the number of genes in the module. The columns represent in an analogous way the modules for the ECLIPSE modules from [31] (recall that we filtered the genes as described in Materials and methods). The number in each cell gives the number of genes common to the modules in the corresponding row and column; the heatmap colors represent the −log10 transformed p-values (truncated at 50), which are based on Fisher’s exact test. Note that the module colors have no meaning, they simply represent consensus modules or ECLIPSE’s modules. (TIFF) [file pone.0185682.s006.tiff]

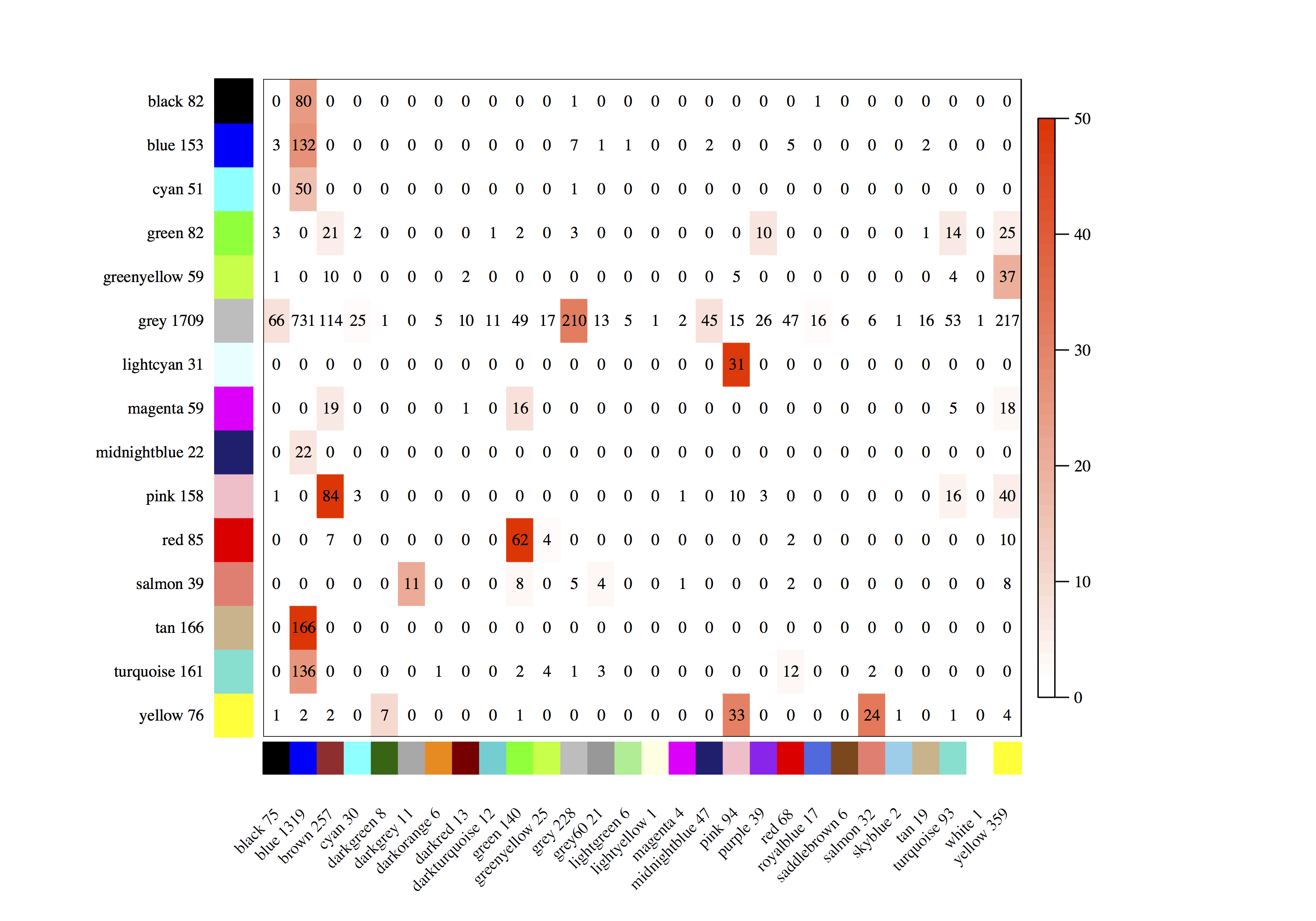

Supplement: S7 Fig — This overlap analysis is performed analogously to the one in S6 Fig, but with the TESRA cohort instead of the ECLIPSE cohort. See also Materials and methods for a filtering of the genes. (TIFF) [file pone.0185682.s007.tiff]
