# Supplementary material for: Meta-analysis of peripheral blood gene expression modules for COPD phenotypes
Source: PLoS One. 2017 Oct 9;12(10):e0185682. doi: 10.1371/journal.pone.0185682 (PMC5633174; doi:10.1371/journal.pone.0185682)
Supplement: S1 Table — (PDF) [file pone.0185682.s011.pdf]

S1 Table.

Outcome	Model (R function)	Covariates Included in Model*
$FEV_1\%$	Linear regr. (lm)	Intercept only
FEV_1/FVC	Beta regr. (betareg)	Age, Sex
Frac. Emph.	Beta regr. (betareg)	Age, Sex, $FEV_1\%$, BMI, Smok. Status**

* All models include an intercept term.

** Only COPDGene data is adjusted for smoking status, since only this cohort has current smokers.
