# Supplementary material for: Meta-analysis of peripheral blood gene expression modules for COPD phenotypes
Source: PLoS One. 2017 Oct 9;12(10):e0185682. doi: 10.1371/journal.pone.0185682 (PMC5633174; doi:10.1371/journal.pone.0185682)
Supplement: S2 Table — The structure of this table is the same as of Table 3. The difference is that we considered molecular function GO terms rather than biological processes terms. (PDF) [file pone.0185682.s012.pdf]

S2 Table.

Module	Top Molecular Function	Ref. list 6322 genes	# Hits	Fold Enrich.	p-value	p.adjust
black 164 genes	RNA polym. II basal transcription factor binding (GO:0001091)	5	2	15.42	7.7E-03	4.3E-02
	3-hydroxyacyl-CoA dehydrogenase activity (GO:0003857)	6	2	12.85	1.1E-02	4.3E-02
	DNA replication origin binding (GO:0003688)	6	2	12.85	1.1E-02	4.3E-02
	Tat protein binding (GO:0030957)	6	2	12.85	1.1E-02	4.3E-02
	promoter-specific chromatin binding (GO:1990841)	6	2	12.85	1.1E-02	4.3E-02
	organic cyclic compound binding (GO:0097159)	2519	78	1.19	2.7E-02	4.7E-02
	heterocyclic compound binding (GO:1901363)	2499	77	1.19	3.2E-02	4.7E-02
	ATPase activity (GO:0016887)	197	10	1.96	3.4E-02	4.7E-02
	unfolded protein binding (GO:0051082)	49	4	3.15	4.0E-02	4.7E-02
	nucleic acid binding (GO:0003676)	1758	56	1.23	4.4E-02	4.7E-02
salmon 68 genes	adenylyltransferase activity (GO:0070566)	15	4	24.79	2.3E-05	1.2E-04
	double-stranded RNA binding (GO:0003725)	47	5	9.89	1.6E-04	7.0E-04
	threonine-type endopeptidase activity (GO:0004298)	13	3	21.45	3.9E-04	1.6E-03
	threonine-type peptidase activity (GO:0070003)	13	3	21.45	3.9E-04	1.6E-03
	tumor necrosis factor receptor binding (GO:0005164)	15	3	18.59	6.0E-04	2.3E-03
	RNA polym. II core promoter seq.-spec. DNA bind. (GO:0000979)	21	3	13.28	1.6E-03	4.8E-03
	tumor necrosis factor receptor superfamily binding (GO:0032813)	24	3	11.62	2.3E-03	6.2E-03
	nucleotidyltransferase activity (GO:0016779)	64	4	5.81	5.1E-03	1.1E-02
	zinc ion binding (GO:0008270)	503	12	2.22	7.0E-03	1.3E-02
	transition metal ion binding (GO:0046914)	572	13	2.11	7.4E-03	1.4E-02
red 168 genes	receptor activity (GO:0004872)	252	19	2.84	4.7E-05	1.4E-03
	molecular transducer activity (GO:0060089)	252	19	2.84	4.7E-05	1.4E-03
	monoval. inorg. cation transmemb. transp. activity (GO:0015077)	75	9	4.52	2.0E-04	3.4E-03
	active transmembrane transporter activity (GO:0022804)	106	10	3.55	6.0E-04	6.8E-03
	receptor binding (GO:0005102)	448	24	2.02	8.3E-04	8.3E-03
	actin binding (GO:0003779)	154	12	2.93	9.3E-04	8.8E-03
	hydrogen-exporting ATPase activity (GO:0036442)	19	4	7.92	1.8E-03	1.3E-02
	protein phosphatase 1 binding (GO:0008157)	10	3	11.29	2.5E-03	1.5E-02
	hydrogen ion transmembrane transporter activity (GO:0015078)	51	6	4.43	2.6E-03	1.5E-02
	secondary active transmemb. transporter activity (GO:0015291)	52	6	4.34	2.9E-03	1.6E-02
greenyellow 100 genes	peptidase regulator activity (GO:0061134)	58	6	6.54	3.4E-04	6.6E-03
	endopeptidase inhibitor activity (GO:0004866)	39	5	8.11	4.1E-04	6.6E-03
	peptidase inhibitor activity (GO:0030414)	39	5	8.11	4.1E-04	6.6E-03
	monosaccharide binding (GO:0048029)	24	4	10.54	6.1E-04	7.6E-03
	endopeptidase regulator activity (GO:0061135)	43	5	7.35	6.4E-04	7.6E-03
	carbohydrate binding (GO:0030246)	77	6	4.93	1.5E-03	1.1E-02
	cysteine-type endopeptidase inhibitor activity (GO:0004869)	22	3	8.62	5.3E-03	2.0E-02
	protease binding (GO:0002020)	44	4	5.75	5.4E-03	2.0E-02
	cytokine activity (GO:0005125)	29	3	6.54	1.1E-02	2.6E-02
	antioxidant activity (GO:0016209)	30	3	6.32	1.2E-02	2.7E-02
