# Supplementary material for: Meta-analysis of peripheral blood gene expression modules for COPD phenotypes
Source: PLoS One. 2017 Oct 9;12(10):e0185682. doi: 10.1371/journal.pone.0185682 (PMC5633174; doi:10.1371/journal.pone.0185682)
Supplement: S3 Table — Overrepresentation of cell type specific genes in consensus modules was calculated using Fisher’s exact test. The 11 groups of distinct immune cell types considered consist of eosinophils (eos), basophils/mast cells (mast_baso), dendritic cells (dendr), neutrophils (neut), b-cells, t-cells, NK-cells, t-helper cells (thelp), monocyte Lipopolysaccharides (LPS) day 0 stimulation, monocyte LPS day 1 stimulation, monocyte LPS day 7 stimulation (mono_d0, mono_d1, mono_d7, respectively). (PDF) [file pone.0185682.s013.pdf]

S3 Table.

	mono_d0	mono_d1	mono_d7	NK-cell	t-cell	b-cell	neut	dendr	eos	mast_baso	thelp
turquoise	6.3E-03	2.2E-01	1.7E-01	1.0E+00	1.0E+00	1.0E+00	3.0E-02	2.8E-01	7.4E-01	6.7E-01	1.0E+00
blue	5.9E-01	1.5E-02	6.3E-01	1.0E+00	3.7E-01	6.4E-01	1.0E+00	1.0E+00	1.0E-01	1.0E+00	7.4E-01
midnightblue	1.0E+00	1.0E+00	2.2E-01	1.0E+00	1.0E+00	1.0E+00	2.5E-01	1.0E+00	1.0E+00	1.0E+00	1.0E+00
tan	4.5E-01	8.4E-01	2.4E-03	5.4E-01	1.0E+00	5.4E-01	1.0E+00	1.0E+00	6.4E-02	1.0E+00	9.6E-01
black	1.0E+00	7.0E-01	1.0E+00	9.1E-03	4.9E-01	1.0E+00	1.0E+00	1.0E+00	1.0E+00	1.0E+00	8.5E-02
cyan	1.0E+00	2.3E-01	1.0E+00	1.0E+00	1.0E+00	1.0E+00	1.0E+00	1.0E+00	6.0E-02	1.0E+00	1.0E+00
salmon	1.0E+00	3.2E-01	1.0E+00	1.0E+00	1.0E+00	1.0E+00	1.0E+00	2.1E-02	1.0E+00	1.0E+00	1.0E+00
lightcyan	1.0E+00	6.5E-01	1.0E+00	1.0E+00	4.5E-01	3.6E-01	6.8E-01	1.0E+00	2.2E-01	1.0E+00	2.0E-01
green	3.3E-01	7.7E-01	7.4E-01	1.7E-02	5.6E-01	4.6E-01	1.0E+00	1.0E+00	1.0E+00	1.0E+00	6.9E-01
pink	5.0E-01	5.5E-01	1.0E+00	1.0E+00	1.0E+00	1.0E+00	1.0E+00	4.2E-01	1.0E+00	1.0E+00	9.9E-02
magenta	1.0E+00	4.8E-01	4.6E-01	1.0E+00	1.0E+00	1.0E+00	1.8E-02	1.0E+00	1.0E+00	1.0E+00	1.0E+00
yellow	1.0E+00	8.0E-01	4.1E-01	1.0E+00	5.9E-01	4.9E-01	4.9E-01	2.8E-01	3.2E-01	2.7E-01	9.4E-01
red	4.5E-01	2.4E-01	1.0E+00	1.0E+00	1.0E+00	1.0E+00	7.9E-06	1.0E+00	1.0E+00	1.0E+00	1.0E+00
greenyellow	1.3E-03	1.7E-01	1.4E-01	1.0E+00	1.0E+00	1.0E+00	1.0E+00	2.0E-01	1.0E+00	1.0E+00	1.0E+00
grey	1.0E+00	1.0E+00	9.7E-01	2.9E-01	1.8E-02	6.8E-02	9.3E-01	4.1E-01	9.8E-01	1.4E-01	2.7E-05
